# Laser Light Treatment Improves the Mineral Composition, Essential Oil Production and Antimicrobial Activity of Mycorrhizal Treated *Pelargonium* *graveolens*

**DOI:** 10.3390/molecules27061752

**Published:** 2022-03-08

**Authors:** Mohammad K. Okla, Samina Rubnawaz, Turki M. Dawoud, Saud Al-Amri, Mohamed A. El-Tayeb, Mostafa A. Abdel-Maksoud, Nosheen Akhtar, Ahlem Zrig, Gehad AbdElgayed, Hamada AbdElgawad

**Affiliations:** 1 Botany and Microbiology Department, College of Science, King Saud University, Riyadh 11451, Saudi Arabia; tdawoud@ksu.edu.sa (T.M.D.); saualamri@ksu.edu.sa (S.A.-A.); mali5@ksu.edu.sa (M.A.E.-T.); 2 Department of Biochemistry, Faculty of Biological Sciences, Quaid Azam University, Islamabad 45320, Pakistan; 3 Zoology Department, College of Science, King Saud University, Riyadh 11451, Saudi Arabia; harrany@gmail.com; 4 Department of Biological Sciences, National University of Medical Sciences, Rawalpindi 46000, Pakistan; nosheenakhtar@numspak.edu.pk; 5 Research Unit of Biodiversity and Valorization of Bioresources in Arid Zones, Faculty of Sciences of Gabès-City Erriadh, Zrig, Gabes 6072, Tunisia; ahlem18zrig@yahoo.fr; 6 Integrated Molecular Plant Physiology Research, Department of Biology, University of Antwerp, 2020 Antwerpen, Belgium; gehad.hegazygadabdelgayed@uantwerpen.be; 7 Department of Botany and Microbiology, Faculty of Science, Beni-Suef University, Beni-Suef 62511, Egypt; hamada.abdelgawad@uantwerpen.be

**Keywords:** mycorrhizal inoculum, laser light, synergistic effect, essential oils, antimicrobial activity

## Abstract

*Pelargonium graveolens*, rose-scented geranium, is commonly used in the perfume industry. *P. graveolens* is enriched with essential oils, phenolics, flavonoids, which account for its tremendous biological activities. Laser light treatment and arbuscular mycorrhizal fungi (AMF) inoculation can further enhance the phytochemical content in a significant manner. In this study, we aimed to explore the synergistic impact of these two factors on *P. graveolens*. For this, we used four groups of surface-sterilized seeds: (1) control group1 (non-irradiated; non-colonized group); (2) control group2 (mycorrhizal colonized group); (3) helium-neon (He-Ne) laser-irradiated group; (4) mycorrhizal colonization coupled with He-Ne laser-irradiation group. Treated seeds were growing in artificial soil inculcated with *Rhizophagus irregularis* MUCL 41833, in a climate-controlled chamber. After 6 weeks, *P. graveolens* plants were checked for their phytochemical content and antibacterial potential. Laser light application improved the mycorrhizal colonization in *P. graveolens* plants which subsequently increased biomass accumulation, minerals uptake, and biological value of *P. graveolens*. The increase in the biological value was evident by the increase in the essential oils production. The concomitant application of laser light and mycorrhizal colonization also boosted the antimicrobial activity of *P. graveolens*. These results suggest that AMF co-treatment with laser light could be used as a promising approach to enhance the metabolic content and yield of *P. graveolens* for industrial and pharmaceutical use.

## 1. Introduction

The worldwide interest in the use of medicinal plants as a therapeutic agent has been arising due to their ease of availability and administration, cost-effectiveness, and perhaps fewer side effects than synthetic drugs. Medicinal plants are a rich source of active ingredients known as primary and secondary metabolites. Many ethnobotanical surveys have also proven the therapeutic benefits of plant-based metabolites [1,2]. *Pelargonium graveolens* (L.) Herit. is one of the medicinal herbs which belongs to the Geraniaceae family and is commonly known as rose-scented geranium. *P. graveolens* is known for its aromatic properties and rich content of terpenoid-essential oil, phenolics, and flavonoids. Therefore, it has been utilized in the flavor, perfume, and fragrance industries [3,4]. Moreover, essential oils extracted from the aqueous extract of *P. graveolens* exhibit in vitro antimicrobial activity, especially against *Bacillus cereus*, *Bacillus subtilis*, and *Staphylococcus aureus* [5]. Geraniol, the major rose oil constituent (about 28%), and rose geranium essential oil constituent (10%) are demonstrated to have antimicrobial activity against different microorganisms [6]. Moreover, a recent study by Guimaraes et al. (2019) [7] showed that geraniol and citronellol are fast-acting compounds that inactivate several microbial species such as *Escherichia* *coli and Salmonella* *typhimurium* by inducing loss of cellular membrane integrity or function. In this regard, essential oil acts on microbial cells and causes structural and functional damage to their membranes [8] that results in increased permeability. These essential oils have also been introduced as a source of new and safe anti-inflammatory drugs [9].

Furthermore, phytochemicals mediated synthesis of various nanoparticles has shown tremendous antimicrobial activity which may lead to novel treatments for cutaneous pathogens. Precisely engineered nanomaterial-based drug delivery systems can easily enter microorganisms and kill or slow bacterial growth locally with the fewest toxic effects to surrounding tissue [10]. Interestingly, the green synthesis of silver and zinc oxide (ZnO) nanoparticles from green tea leaves has been emerging as an eco-friendly and cost-effective antibacterial and chemopreventive approach [11,12].

Nowadays, different elicitation strategies have been used to improve the phytochemical content and their bioactivity in different plants. Laser irradiation, a physical elicitor, has shown positive bio stimulatory properties that result in the increasing production and yield of many plant species [13]. Laser light application is considered an acceptable mechanism in large-scale agricultural areas as it ensures environmental safety [14,15]. Laser stimulation of seeds causes the absorption and storage of light energy in the plant seeds and leaves. In seeds, the absorbed light energy is transformed into chemical energy before storage. Irradiation with helium-neon (He-Ne) laser light increases the energy potential of seeds, enhancing the cell division and germination capacity and strengthening the plant development [16,17]. Long-term irradiation with He-Ne laser light can stimulate morphological and physiological changes including the increased organ mass, increased chlorophyll content in leaves which subsequently affects the photosynthetic activity in seedlings and plants [13]. In our previous experiment, we illustrated that laser light improves the nutritional value, antioxidant capacity, and anti-inflammatory activity of flavonoid-rich buckwheat seedlings [18]. Laser light induction also improved the biomass production, photosynthetic rate, chemical composition, and biological activities of lemongrass sprouts [19]. Previous studies depict that laser light can manipulate and fine-tune certain plant metabolic pathways, thus enhancing biomass production in a specie and dose-specific manner [20].

Many plant roots have a mutualistic symbiotic relation with Arbuscular mycorrhizal fungi (AMF) which belongs to the subphylum, Glomeromycotina [21]. These AMF can provide numerous benefits to plants, including enhanced nutrient uptake, better growth, and resistance to abiotic stresses, such as salinity [22], high temperature [23], and drought stress [24]. Roots colonization with AMF produces several metabolic changes including the activation of the Krebs cycle and plastid biosynthetic pathways. This leads to a marked surge in metabolic activity and accumulation of primary and secondary metabolic products [25]. Previously, it was found that AMF together with *B. subtilis* had a synergistic effect on the biomass and essential oil yield of *P. graveolens* [26]. Moreover, mycorrhizal inoculation ameliorated the antioxidant activity and secondary metabolites in *P. graveolens* [21].

These mycorrhizal-associated positive effects on the plant bioactive yield besides the bio stimulatory effects of laser light application were the primary factors for the current study design. It is worthwhile to note that there is no report to assess the impact of laser light on *P. graveolens*. Therefore, we aimed to investigate the possible synergistic effects of laser light application on the mycorrhizal treated *P. graveolens* in terms of its mineral composition, essential oil production, and antimicrobial activity.

## 2. Results

### 2.1. Laser Improved Mycorrhizal Colonization in P. graveolens Plants

The application of laser light in the current study has significantly improved the mycorrhiza colonization in *P. graveolens* as represented in Table 1. Six weeks after AM fungal inoculation, no signs of AMF were detected in the roots obtained from non-inoculated plants. The rate of root colonization in the laser-treated group was increased by about 29% compared to that in the control. Moreover, the hyphal length of mycorrhiza has been increased by about 48% in the laser-treated group in comparison to the control group. Additionally, the number of arbuscules has been increased in the laser-treated group by about 30% in comparison to the control group.

### 2.2. Improved Colonization in Plant-Initiated from Laser Light Treated Seeds Increased Biomass Accumulation and Biological Value of P. graveolens

**Figure 1:**
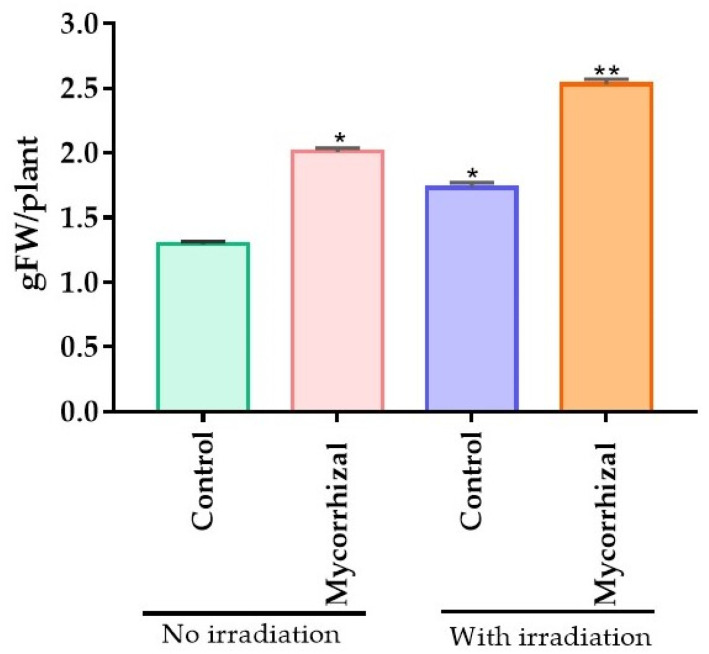
Impact of laser light and mycorrhizal co-treatment on plant biomass. Values are presented as mean ± SD whereas, * = *p* < 0.05, ** = *p* < 0.01.

The bio stimulatory effects of laser light application in mycorrhiza-colonized *P. graveolens* were investigated. As illustrated in Figure 1, the plant biomass (g fresh weight/plant) was significantly increased (*p* < 0.05) in the laser-treated mycorrhiza-colonized group (2.53 ± 0.1 g FW/plant) of *P. graveolens* compared to the laser non-treated group (2.01 ± 0.1 g FW/plant) and control plant group (*p* < 0.01). Interestingly, the laser light irradiation also displayed improved biomass in the control group (1.73 ± 0.1 g FW/plant) when compared to their non-irradiated normal control counterparts (1.29 ± 0.1 g FW/plant).

**Figure 2:**
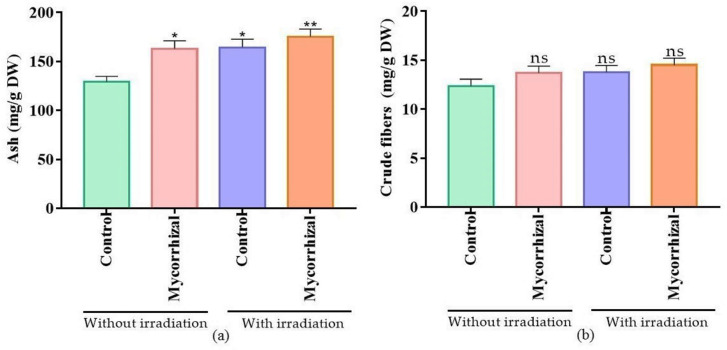
Effect of laser light and mycorrhizal co-treatment on plant (**a**) ash and (**b**) crude fibers content. Values are presented as mean ± SD whereas, * = *p* < 0.05, ** = *p* < 0.01, ns = non-significant.

Primary and secondary metabolic contents were highest in the laser and mycorrhizal cotreated groups (Table 2). This coupled treatment almost doubled the total phenolics and flavonoids content (21.5 ± 1 and 1.2 ± 0.1 mg/g DW, respectively) when compared to the untreated control plants (11.07 ± 1.2 and 0.53 ± 0.1 mg/g DW, respectively). Moreover, total protein, total sugar, and total alkaloid contents were also significantly higher (*p* < 0.05) in the laser and mycorrhizal cotreated group than the rest of the treatment groups. Similarly, treated samples produced a significantly higher amount of ash (*p* < 0.05). However, crude fiber level was somewhat similar in all samples (Figure 2a,b). Overall, different metabolites ranked in the order of laser and mycorrhizal cotreated group > laser-treated control > only mycorrhizal treated group > untreated control group with increasing nutrient content.

### 2.3. Mycorrhiza Induced Mineral Uptake by P. graveolens Is Improved by Laser Light Treatment

Table 3 shows the impact of mycorrhizal and laser light treatment on the mineral content of *P. graveolens*. Laser light combined with mycorrhizal treatment significantly (*p* < 0.05) induced the accumulation of different minerals in *P. graveolens*. Meanwhile, less significant changes were observed in laser light treated plants compared to their untreated controls. Just like the nutrient content, laser light combined with mycorrhizal treatments almost doubled the levels of potassium (K), phosphorus (P), calcium (Ca), magnesium (Mg), and iron (Fe). Similarly, laser light and mycorrhiza alone also improved the mineral uptake when compared to untreated control plants. However, Zinc (Zn) remained unchanged in treated and untreated plants.

### 2.4. Co-Application of Mycorrhiza and Laser Light Treatment Induced the Production of Essential Oils and Their Precursors

Here, 28 essential oils and 3 important precursors (phenylalanine, cinnamic acid, and shikimic acid) were found in all samples. Among identified essential oils, geraniol and beta-citronellol were most predominant in all plants. Whereas shikimic acid was the most abundant essential oil precursor (72.9 ± 2.5–90.4 ± 1.4%) in treated and untreated samples. Generally, mycorrhizal-laser light cotreatment showed the most significant (*p* < 0.05) impact on the oil yield and its content. However, laser light and mycorrhizal alone treatments showed somewhat similar oil content when compared to untreated controls. Table 4 shows that the percentage of α-pinene, β-phellandrene, citronellyl formate, geraniol, geraniol formate, linalool, Cis-β-ocimene, I-Menthone, nerol, beta-bourbonene, beta-cubebene, z-citral, trans-caryophyllene, isoledene, ledene, neryl acetate, geranyl tiglate, and mintsulfide was higher in laser-treated group than the control. Surprisingly, all the treatments reduced myrcene content (~0.19%) when compared to untreated control plants (0.33%). Overall, laser light treatment displayed a more pronounced impact on the essential oil content in *P. graveolens*.

### 2.5. Laser Light Boosted the Antibacterial Properties of Mycorrhiza Inoculated P. graveolens Plant

Both gram-positive and negative bacterial strains were used to evaluate the antibacterial activity of *P. graveolens* through the disc diffusion method. Among all the tested bacterial strains, *P. graveolens* showed maximum growth inhibition of *E. coli* (gram-negative) followed by *Streptococcus salivarius* (gram-positive). Yet again, the laser-mycorrhizal coupled treatment group had the largest zones of inhibition (23.54 ± 1.1–35.31 ± 1.2 mm) against all bacterial strains (Table 5). In general, different plant samples followed the pattern of laser light and mycorrhizal group > mycorrhiza treated group > laser light control group > and untreated control group for antibacterial activity.

## 3. Discussion

In the present study, we investigated the synergistic effect of laser light and mycorrhizal treatment on the phytochemical content of *P. graveolens*. For this, the *P. graveolens* sterilized seeds were co-cultivated with *Rhizophagus irregularis* and/or He-Ne laser light in a controlled environment. These treated *P. graveolens* samples were compared with untreated control *P. graveolens* for their biomass, mineral content, nutritional value, and antibacterial activity.

Here, laser light treatment significantly improved the mycorrhizal root colonization, hyphal length, and the number of arbuscular roots when compared with untreated control roots. Laser light induction along with mycorrhizal treatment also improved biomass production. Previously, Alam et al. (2011) [26] also reported that mycorrhizal treatment increased the growth and biomass in rose-scented geranium. The synergistic effects of laser light application could be understood in the light of the postulation that the energy from laser light can stimulate the intensity of the transmembrane electrochemical proton gradient in mitochondria and cell proliferation and produce morphological changes in cells and organisms [27]. It was found that some fungi that have been grown in in vitro culture, have shown an increased rate of cellular bioenergetic processes after exposure to laser light of a specific wavelength [28]. Moreover, plant macromolecules absorb laser light that triggers photosynthetic activity, resulting in increased growth, fresh weight, and biomass [29]. Our recent studies have augmented this postulation whereas laser light application was able to improve the nutritional value, antioxidant capacity, and anti-inflammatory activity of flavonoid-rich buckwheat sprouts and lemongrass sprouts [19].

Increased biomass production could be correlated with the enhanced primary and secondary metabolic content in treated *P. graveolens* plants. We observed enhanced amounts of protein, sugar (primary metabolites), phenolics, flavonoids, and alkaloids (secondary metabolites) in the laser-mycorrhizal treated plants. The laser-light-induced enhanced photosynthesis can mediate the biosynthetic pathways resulting in an increased sugar content [30]. The surplus photosynthetic sugar can generate the cellular energy required for the biosynthesis of primary and secondary metabolites [31] and/or used by AMP for their energy metabolism and growth [32]. Inoculation of olive trees and chamomile with *R. irregularis* significantly improved the accumulation of phenolic and flavonoids content in fungal infected plants [33,34]. Phenolics and flavonoids are the most abundant antioxidants involved in plant defense mechanisms. Several reports suggest the AMP induces the accumulation of Phenylalanine ammonia-lyase (PAL) in the infected roots. PAL is the key enzyme regulating the phenolic biosynthetic pathway. This accounts for higher levels of phenolics in AMP-associated plants [35]. In return, the phenolics and flavonoids exert a positive impact on hyphal growth and arbuscules development during symbiosis [36].

Minerals are essential elements to regulate the proper growth and development of plants. Under field conditions, the unavailability of adequate levels of these minerals can impede plant growth [37]. The previous meta-analysis confirms that AMF increases the soil enzyme activity that releases soil nutrients required for plant growth [38]. Moreover, AMF also inhibits the plant nutrient leaching into the soil thus mitigating the salt/drought stress and maintaining the ion balance [39]. In this study, an enhanced mineral uptake was observed in mycorrhiza and laser-treated plants than untreated control plants. Shao et al. (2018) also reported significantly higher leaf N, P, K, Ca, Mg, Zn, and Mn contents in AMF-inoculated tea plants than in non-AMF-inoculated ones [40]. Similarly, AMP inoculation alleviated the drought stress in *P. graveolens* and improved its metabolic content [14]. Many researchers have reported the enhanced uptake of N, P, and K by AMF-inoculated plants under saline conditions [41,42]. The higher mineral uptake by AMP-inoculated plants, likely results from the extra-radical mycorrhizal hyphae that extend beyond the roots and exploit nutrient-filled soil [43]. Besides increasing the absorption area of the roots, AMF also releases important chemical compounds such as glomalin, a glycoprotein secreted by hyphae and spores of AMF. Glomalin in the soil helps the uptake of nutrients such as Fe and P that are difficult to dissolve [44].

On the other hand, several studies suggest that nutrient absorption and utilization by plants could be modulated according to the light source and the period of duration to this source. Several studies have been uncovered the relationship between light perception and nutrient uptake [30,45]. In particular, laser light application has been proven to improve the nutritional value of many plant species [46,47]. As a result, the combination of mycorrhizal colonization and laser application in the current study has shown a significant synergistic effect on the minerals uptake by *P. graveolens*

The combination of plant essential oils with synthetic drugs offers a promising approach for producing new antimicrobial drugs [48]. *Pelargonium* genus produces the top 20 essential oils in the world, well known for their antimicrobial, anti-inflammatory, and spasmolytic properties [49,50]. In the present study, GC-MS revealed the presence of 28 essential oils in varying levels. Among the detected essential oils, geraniol and beta-citronellol were most abundant in all samples. Previous literature shows that *P. graveolens* essential oil is an enriched source of citronellol and geraniol. It is commonly known as rose geranium oil and is extensively used in perfumes and skincare products [51]. Geranium oils have gained worldwide acceptance in the food industry since they have been recognized as safe products and approved by the American Food and Drug Administration (FDA) for food use [50].

Earlier, laser light improved the levels of essential oils and their precursors in anise [52] which supports our results. Moreover, Balkhyour et al. (2021) [53] reported an induced accumulation of essential oils, phenylalanine, and cinnamic acid in He-Ne laser-treated ajwain seedlings, which is also in agreement with our findings. AMF colonization differentially improved the essential oils of oregano, mint, dill, and coriander [54,55].

These improved essential oils could account for significant antibacterial activities of *P. graveolens* in this study. Several authors have been able to demonstrate the broad-spectrum antibacterial activity of essential oils and plant extracts including rosemary, oregano, basil, tea tree, and myrtle [56,57,58,59] against the bacterial strains under consideration in the present study. Moreover, Hsouna et al. (2012), Rosato et al. (2007), and Ghannadi et al. (2012) reported the pronounced antibacterial activity of *P. graveolens* essential oils with an almost similar chemical profile [5,60,61]. Generally, gram-negative bacteria show high resistance against plant essential oils due to the presence of a hydrophilic outer membrane containing a hydrophilic polysaccharide chain, which acts as a barrier to a hydrophobic essential oil [62]. Contrarily, we observed that *E. coli* (gram-negative) was most susceptible against the laser light and mycorrhizal treated plants followed by *S. salivarius* (gram-negative). Previously, parsley and lovage essential oils demonstrated similar activity against gram-positive and negative bacteria [63]. This can be attributed to the antibacterial potential of geraniol and beta-citronellol (alcohol) in *P. graveolens* plants and part to the metabolic byproducts produced by AMF which impart antioxidant, antiviral, and antibacterial effects [64]. Previous reports have illustrated the antimicrobial activity of narrow-wavelength light, which eliminates a range of common pathogens by inducing oxidative stress that ultimately can kill microbial pathogens [65].

Consequently, the observed antibacterial activities show that laser light and mycorrhizal co-treated *P. graveolens* plants can be ideal candidates against drug-resistant bacterial strains. However, further studies are needed to explore the efficiency of suitable concentration and safety of essential oils as a potential therapeutic agent.

## 4. Materials and Methods

### 4.1. AMF Symbiont

A pure commercial inoculum of *R. irregularis* (Błaszk., Wubet, Renker & Buscot) C. Walker & A. Schüßler (MUCL 41833) was obtained from *Glomeromycota* in vitro collection (GINCO) in a pot (25 × 15 cm). Considering the bibliography, *R. irregularis* is the most popular species, it is easy to propagate in pot culture in association with different host plants. It is also well known for its growth-promoting effect on plants. AMF spores were grown for 6 months on the roots of *Sorghum sudanenses* Pers. The mycorrhizal inoculum was added as 5 g of trapped soil per plant or pot (~50 spores/g soil). The inoculum was inserted before sowing, at a depth of 6 cm below the surface, thus producing mycorrhizal treatment. The controlled plots received equal amounts of autoclaved inoculum to supply equivalent nutrients aside from mycorrhizal spores.

### 4.2. Experimental Setup

A homogenous lot of seeds were surface sterilized in 5% (*v/v*) of sodium hypochlorite for 20 min. Seeds of *P. graveolens* were soaked for 2 h in distilled H_2_O before laser illumination using a laser equipment system (DMC Equipment Ltd. Firenze, Italy). Four groups of seeds were used, each group contained 100 seeds: (1) control group1 (non-irradiated; non-colonized group); (2) control group2 (mycorrhizal colonized group); (3) helium-neon (He-Ne) laser-irradiated group; (4) mycorrhizal colonization coupled with He-Ne laser-irradiation group. *P. graveolens* seeds were irradiated by He-Ne laser (632 nm, 5 mW) with a beam diameter of 1 mm at 500 mJ energy for 5 min from the side of the embryonic area. These conditions were selected following a preliminary experiment for detecting the most optimum laser treatment conditions based on the increase in the growth of *P. graveolens* species to laser in terms of fresh weight. The laser was applied perpendicularly at the distance of 12 cm between the laser source and the seeds.

Treated seeds were growing in artificial soil containing sterilized sand (70%), dry clay (8%), silt (15%), sphagnum peat (10%), organic matter (5.4%), and several minerals such as N (1.2 g/kg), P (0.17 g/kg), K (5.9 g/kg), Ca (79 g/kg), Mg (1.5 g/kg), (9 cmol/kg) cation exchange capacity, 68% soil water capacity. The artificial soil was inculcated with *R. irregularis* MUCL 41833. The control treatments were represented by non-laser and inoculated soil. Plant seeds were grown in a custom build climate-controlled chamber at 21/18 °C in a 16/8 h day/night photoperiod (150 μmol PAR m^−2^ s^−1^, 60% humidity) and exposed to different arsenate (AsO_4_^3−^) soil concentrations (control: 0 mg kg^−1^; severe: 100 mg kg^−1^). The soil water content was kept at 60% throughout the experiment. After 6 weeks of growth, the rhizosphere soil and shoots were collected and aliquoted for further analysis. The fresh weight of roots and shoots was determined.

### 4.3. AMF Growth Analysis

The roots for each treatment were washed, cut into segments, and cleaned with KOH solution. Then, the roots (30 root fragments total) were stained by trypan blue in lacto-glycerol and were examined with a stereomicroscope [66]. The colonization was calculated by using the gridline intersect method and arbuscule abundance in the root system were calculated by the number of arbuscules cm^−1^ root [67]. The hyphal length was determined in rhizosphere and hydrosphere soils by the method of Andrade et al. (1997) [68].

### 4.4. Elemental Analysis

Elemental analysis was done by using inductively coupled plasma (ICP-MS, Finnigan Element XR, Scientific, Bremen, Germany) [69].

### 4.5. Determination of the Nutritional Value

To give insights into the food functional value of *P. graveolens* samples, the levels of amino acids, organic acids, fatty acids, minerals, and vitamins were evaluated as described below.

#### Proximate Composition Analysis

Carbohydrates were extracted by boiling a known weight *P. graveolens* plant tissue in 1 N HCl for 2 h. The extract was cooled and centrifuged at 6000× *g* for 15 min. The supernatant was neutralized with 1 N NaOH and made up to a known volume with distilled water. The concentrations were evaluated following Nelson’s method [70] from each sample. Protein concentration was extracted from 0.5 gFW in 1 M NaOH (5 mL) at 37 °C for 60 min and the soluble material was analyzed for total protein. The concentrations were measured following the Folin-Lowry methods, the absorbance of standards was measured at 660 nm against blank. Total lipids were assessed based on Folch modified by Bligh and Dyer (1959) [71], where the plant samples were homogenized in a mixture of chloroform/ methanol (2:1, *v/v*). Then, centrifuged (15 min, 3000× *g*). The pellets were re-dissolved in a mixture of toluene/ethanol (4/1, *v/v*). Afterward, the extract was mixed with a saline solution. The extracted lipids were concentrated, and the total lipid content was calculated. Crude fibers were extracted and measured according to AOAC (1990) [72]. Samples were gelatinized with a heat-stable alpha-amylase (pH 6, 100 °C, 30 min), and then enzymatically digested with protease (pH 7.5, 60 °C, 30 min) and amyloglucosidase (pH 6, 0 °C, 30 min) to remove undesirable components. Precipitation of Fibers was done with ethanol, then the residue was weighed.

### 4.6. Determination of Essential Oils

*P. graveolens* plants were dried at room temperature and were used for essential oils extraction. The dried plants were exposed to steam distillation for 4 h using a Clevenger instrument. The levels of essential oils in plants were measured by GC/MS as described by Hassanpour et al. (2014) [73]. A gas chromatograph HP-5 (Crosslinked 5% PH ME Siloxane, 15 m × 0.53 mm × 1.5 μm film) column was used for separation at a helium flow rate 2 mL min^−1^, injector temperature 220 °C and detector temperature 240 °C using temperature program 60 °C, 40 °C min^−1^ up to 220 °C, 2 min at 220 °C. Portions of 2 μL of the essential oil (dissolved in *n-hexane*) were injected into the used analytical column. Identification of oil components was achieved based on their retention indices and by comparison of their mass spectral fragmentation patterns (NIST) database. The concentrations of essential oil, as a percentage (%), were calculated.

### 4.7. Antibacterial Activity

The antibacterial activity of the tested plants was evaluated against *B. subtilis*, *S. salivarius*, *E. coli*, *Pseudomonas aeruginosa*, *Sarcina lutea*, and *S. aureus* by the application of the Disc diffusion method [74], using 100 μL of suspension containing 10^8^ CFU/mL of bacteria spread on Muller Hinton agar. The antibacterial activity was indicated based on the inhibition zone, determined by the Vernier caliper.

### 4.8. Statistical Analysis

Statistical analysis was performed using the SPSS statistical package (SPSS Inc., Chicago, IL, USA). One-way analysis of variance (ANOVA) was applied to all data. Tukey’s Test (*p* < 0.05) was carried out as the post-hoc test for mean separations. Each experiment was replicated at least three times (*n* = 3).

## 5. Conclusions

Both the application of laser light and mycorrhizal colonization have several beneficial effects on the biological value of many plants. However, laser stimulation coupled with mycorrhizal inoculation significantly improved the plant biomass and its nutrient content in *P. graveolens*. The treated samples also showed improved essential oils and primary and secondary metabolites when compared to untreated control plants. Moreover, laser light and mycorrhizal co-treatment boosted the antibacterial activities of *P. graveolens* against Gram-positive and negative bacteria. Thus, this study supports the co-application of laser light and AMF as an advantageous approach for increasing the nutritional and health-promoting effects of *P. graveolens*.

## Figures and Tables

**Table 1:** Effect of irradiation and mycorrhizal inoculation on growth parameters.

Growth Parameters	No Irradiation		Laser Irradiation	
	Control	Mycorrhizal	Control	Mycorrhizal
Colonization (% root)	0 ± 0 ^a^	38.7 ± 3.1 ^b^	0 ± 0 ^a^	49.3 ± 3.7 ^c^
Hyphal length (cm/g soil)	0 ± 0 ^a^	13.1 ± 1.4 ^b^	0 ± 0 ^a^	19.4 ± 0.7 ^c^
Number of arbuscules/cm root	0 ± 0 ^a^	5.2 ± 0.2 ^b^	0 ± 0 ^a^	6.8 ± 0.3 ^c^

Values are presented as mean ± SD whereas, different superscripts (a–c) show statistical significance among the data at *p* < 0.05.

**Table 2:** Biological content of *P. graveolens*.

Metabolites (mg/g DW)	No Irradiation		Laser Irradiation	
	Control	Mycorrhizal	Control	Mycorrhizal
Total protein	148.68 ± 8 ^a^	158.3 ± 9 ^b^	163.5 ± 11.8 ^b^	186.5 ± 6.5 ^c^
Total sugar	59.68 ± 2.6 ^a^	61.7 ± 3.6 ^a^	64.1 ± 3.9 ^a^	76.4 ± 3.1 ^b^
Total phenols	11.07 ± 1.2 ^a^	14.5 ± 1 ^b^	17.5 ± 1 ^a^	21.5 ± 1 ^c^
Total flavonoids	0.53 ± 0.1 ^a^	0.8 ± 0.1 ^b^	0.8 ± 0.1 ^b^	1.2 ± 0.1 ^c^
Total alkaloid	18.5 ± 1.6 ^a^	26.3 ± 2 ^b^	19.4 ± 2.4 ^a^	24.1 ± 2.9 ^b^

Values are presented as mean ± SD whereas, different superscripts (a–c) show statistical significance among the data at *p* < 0.05.

**Table 3:** Effect of mycorrhiza and laser light on mineral uptake by *P. graveolens*.

Minerals (mg/g DW)	No Irradiation		Laser Irradiation	
	Control	Mycorrhizal	Control	Mycorrhizal
K	12.2 ± 0.7 ^a^	17.7 ± 0.5 ^b^	16.4 ± 0.8 ^b^	26.9 ± 0.7 ^c^
P	4.57 ± 0.4 ^a^	6.67 ± 0.3 ^b^	5.89 ± 0.6 ^a^	8.16 ± 0.3 ^c^
Ca	2.32 ± 0.3 ^a^	3.4 ± 0.2 ^ab^	2.95 ± 0.2 ^a^	4.3 ± 0.3 ^b^
Mg	1.81 ± 0.3 ^a^	2.7 ± 0.2 ^ab^	2 ± 0.3 ^a^	3.47 ± 0.3 ^b^
Na	0.39 ± 0.1 ^a^	0.61 ± 0.1 ^b^	0.53 ± 0.1 ^ab^	0.65 ± 0.1 ^b^
Fe	0.15 ± 0 ^a^	0.23 ± 0 ^ab^	0.21 ± 0 ^a^	0.27 ± 0 ^bc^
Zn	0.04 ± 0 ^a^	0.06 ± 0 ^a^	0.06 ± 0 ^a^	0.05 ± 0 ^a^

Values are presented as mean ± SD whereas, different superscripts (a–c) show statistical significance among the data at *p* < 0.05.

**Table 4:** Essential oil content in control and treated *P. graveolens*.

Parameters	No Irradiation		Laser Irradiation	
	Control	Mycorrhizal	Control	Mycorrhizal
***Oil yield (%)***	12.8 ± 0.3 ^a^	12.7 ± 0.5 ^a^	13.4 ± 0.5 ^a^	16.8 ± 0.4 ^b^
***Essential oil (% dry weight)***	3.1 ± 0.1 ^a^	3.48 ± 0.2 ^a^	3.8 ± 0.2 ^a^	4.6 ± 0.1 ^a^
***Total essential oil (%)***				
Beta-Citronellol	16.4 ± 0.1 ^a^	17.1 ± 0.1 ^a^	18.3 ± 0.1 ^b^	20.4 ± 0.1 ^b^
Citronellyl formate	7.3 ± 0.6 ^a^	7.8 ± 0.8 ^a^	8.9 ± 0.9 ^a^	9.3 ± 0.7 ^b^
Citronellyl propionate	0.09 ± 0 ^a^	0.08 ± 0 ^a^	0.09 ± 0 ^a^	0.14 ± 0 ^b^
Geraniol	20.1 ± 0.8 ^a^	27.7 ± 1 ^b^	22.6 ± 1.1 ^b^	34.8 ± 0.9 ^c^
Geraniol formate	7.05 ± 0.7 ^a^	8.73 ± 1 ^a^	7.76 ± 1 ^a^	9.8 ± 0.8 ^b^
α-pinene	1.04 ± 0.1 ^a^	1.68 ± 0.1 ^a^	1.68 ± 0.1 ^a^	1.7 ± 0.1 ^a^
Limonene	0.17 ± 0 ^a^	0.16 ± 0 ^a^	0.15 ± 0 ^a^	0.26 ± 0 ^b^
Myrcene	0.33 ± 0 ^a^	0.19 ± 0 ^a^	0.19 ± 0 ^a^	0.18 ± 0 ^a^
Linalool	4.04 ± 0.1 ^a^	4.99 ± 0.2 ^b^	4.17 ± 0.2 ^a^	5.33 ± 0.1 ^b^
Cis-β-ocimene	0.24 ± 0.1 ^a^	0.27 ± 0.1 ^a^	0.25 ± 0.1 ^a^	0.31 ± 0.1 ^a^
Sabinene	0.3 ± 0 ^a^	0.5 ± 0 ^b^	0.27 ± 0 ^a^	0.48 ± 0 ^b^
p-cymene	0.57 ± 0 ^a^	0.52 ± 0 ^a^	0.54 ± 0 ^a^	0.8 ± 0 ^b^
Beta-phellandrene	0.7 ± 0.1 ^a^	0.75 ± 0.1 ^a^	0.95 ± 0.1 ^b^	0.91 ± 0.1 ^b^
I-Menthol	0.04 ± 0.1 ^a^	0.13 ± 0.2 ^b^	0.04 ± 0.2 ^a^	0.08 ± 0.1 ^b^
I-Menthone	4.55 ± 0.2 ^a^	5.12 ± 0.2 ^a^	5.36 ± 0.2 ^a^	5.9 ± 0.2 ^a^
Nerol	3.66 ± 0.2 ^a^	4.18 ± 0.3 ^a^	4.31 ± 0.3 ^a^	5.91 ± 0.3 ^b^
Beta-bourbonene	0.75 ± 0.4 ^a^	1.02 ± 0.5 ^a^	0.82 ± 0.5 ^a^	0.93 ± 0.4 ^a^
Beta-cubebene	0.94 ± 0.2 ^a^	1.11 ± 0.3 ^a^	1.55 ± 0.3 ^ab^	2.6 ± 0.2 ^b^
z-citral	0.04 ± 0 ^a^	0.05 ± 0 ^a^	0.05 ± 0 ^a^	0.06 ± 0 ^a^
α-Terpinene	0.04 ± 0 ^a^	0.07 ± 0 ^b^	0.04 ± 0 ^a^	0.085 ± 0 ^b^
Trans-caryophyllene	1.67 ± 0 ^a^	1.84 ± 0 ^a^	1.97 ± 0 ^a^	1.97 ± 0 ^a^
Isoledene	1.4 ± 0 ^a^	2.55 ± 0 ^b^	1.65 ± 0 ^b^	2.95 ± 0 ^b^
Germacrene-D	3.83 ± 0 ^a^	3.48 ± 0 ^a^	3.71 ± 0 ^a^	5.3 ± 0 ^b^
Ledene	3.19 ± 0 ^a^	3.31 ± 0 ^a^	3.53 ± 0 ^a^	3.97 ± 0 ^a^
δ-cadinene	1.83 ± 0 ^a^	1.52 ± 0 ^a^	1.61 ± 0 ^a^	2.7 ± 0 ^b^
Neryl acetate	1.58 ± 0 ^a^	2.75 ± 0.1 ^b^	1.64 ± 0.1 ^a^	2.97 ± 0 ^b^
Geranyl tiglate	1.77 ± 0.1 ^a^	1.98 ± 0.1 ^a^	3.15 ± 0.1 ^b^	3.37 ± 0.1 ^b^
Mintsulfide	2.5 ± 0.7 ^a^	4.62 ± 0.9 ^b^	4.46 ± 1 ^b^	6.12 ± 0.8 ^c^
***Essential oil precursors***				
Phenylalanine	3.37 ± 0.2 ^a^	3.43 ± 0.3 ^a^	3.53 ± 0.3 ^a^	5.41 ± 0.2 ^b^
Cinnamic acid	4.55 ± 0.4 ^a^	7.36 ± 0.5 ^b^	3.32 ± 0.6 ^a^	7.4 ± 0.5 ^b^
Shikimic acid	72.9 ± 2.5 ^a^	87.6 ± 3.4 ^b^	81.21 ± 3.6 ^a^	90.4 ± 1.4 ^b^

Values are presented as mean ± SD whereas, different superscripts (a–c) show statistical significance among the data at *p* < 0.05.

**Table 5:** Antibacterial activities of *P. graveolens*.

Bacterial Specie	No Irradiation		Laser Irradiation	
	Control	Mycorrhizal	Control	Mycorrhizal
	Zones of Inhibition (mm)			
*Bacillus subtilis*	13.2 ± 0.6 ^a^	21 ± 1.3 ^b^	18.1 ± 0.8 ^b^	25.1 ± 1 ^c^
*Streptococcus* *salivarius*	21.8 ± 0.4 ^a^	30 ± 1.4 ^b^	23.54 ± 1 ^a^	32.1 ± 0.9 ^b^
*Escherichia coli*	18.6 ± 0.6 ^a^	25 ± 1.8 ^b^	16.05 ± 0.8 ^a^	35.31 ± 1.2 ^c^
*Pseudomonas aeruginosa*	19.3 ± 1 ^a^	29 ± 1.1 ^b^	17.98 ± 0.9 ^a^	23.54 ± 1 ^a^
*Sarcina lutea*	14.7 ± 0.5 ^a^	23 ± 1.2 ^b^	22.47 ± 1.2 ^b^	31.03 ± 0.9 ^c^
*Staphylococcus aureus*	15.8 ± 1.2 ^a^	22 ± 1 ^b^	14.98 ± 1.8 ^a^	23.54 ± 1.1 ^b^

Values are presented as mean ± SD whereas, different superscripts (a–c) show statistical significance among the data at *p* < 0.05.

## Data Availability

The data that support the findings of this study are available on request from the corresponding author.

## Sample Availability

Samples of the compounds are available from the authors.
